# Clinicopathological significance of ataxia telangiectasia-mutated (ATM) kinase and ataxia telangiectasia-mutated and Rad3-related (ATR) kinase in MYC overexpressed breast cancers

**DOI:** 10.1007/s10549-018-05113-8

**Published:** 2019-02-12

**Authors:** Constantinos Savva, Karen De Souza, Reem Ali, Emad A. Rakha, Andrew R. Green, Srinivasan Madhusudan

**Affiliations:** 1 Department of Oncology, Nottingham University Hospitals, Nottingham, NG5 1PB UK; 2 Translational Oncology, Nottingham Breast Cancer Research Centre, Division of Cancer and Stem Cells, Academic Unit of Oncology, School of Medicine, University of Nottingham, Nottingham, NG51 PB UK; 3 Department of Pathology, Nottingham Breast Cancer Research Centre, Division of Cancer and Stem Cells, School of Medicine, University of Nottingham, Nottingham, NG5 1PB UK

**Keywords:** ATM, ATR, MYC, Breast cancer

## Abstract

**Purpose:**

MYC transcription factor has critical roles in cell growth, proliferation, metabolism, differentiation, transformation and angiogenesis. MYC overexpression is seen in about 15% of breast cancers and linked to aggressive phenotypes. MYC overexpression also induces oxidative stress and replication stress in cells. ATM signalling and ATR-mediated signalling are critical for MYC-induced DNA damage response. Whether ATM and ATR expressions influence clinical outcomes in MYC overexpressed breast cancers is unknown.

**Methods:**

We investigated ATM, ATR and MYC at the transcriptional level [Molecular Taxonomy of Breast Cancer International Consortium cohort (*n* = 1950)] and at the protein level in the Nottingham series comprising 1650 breast tumours. We correlated ATM, ATR and MYC expressions to clinicopathological features and survival outcomes.

**Results:**

In MYC over expressed tumours, high ATR or low ATM levels were associated with aggressive breast cancer features such as higher tumour grade, de-differentiation, pleomorphism, high mitotic index, high-risk Nottingham Prognostic Index, triple negative and basal-like breast cancers (all adjusted *p* values < 0.05). Tumours with low ATM or high ATR levels in conjunction with MYC overexpression also have worse overall breast cancer-specific survival (BCSS) (*p* value < 0.05).

**Conclusions:**

We conclude that ATR/ATM-directed stratification and personalisation of therapy may be feasible in MYC overexpressed breast cancer.

**Electronic supplementary material:**

The online version of this article (10.1007/s10549-018-05113-8) contains supplementary material, which is available to authorized users.

## Background

The c-MYC transcription factor has critical roles in cell growth, proliferation, metabolism, differentiation, transformation and angiogenesis. Overexpression of c-MYC (henceforth MYC) is frequently observed in several solid tumours implying a critical role in tumorigenesis and progression. In addition, MYC overexpression is linked to resistance to chemotherapy and radiotherapy [5, 12, 15, 17, 19, 27, 35, 37]. In breast cancers, MYC gene amplification (15%), MYC mRNA overexpression (22–35%) and MYC protein overexpression (40%) have been reported. MYC overexpression has been linked to specific subtypes of aggressive breast cancers [7, 16, 21, 38].

Overexpression of MYC and resultant oncogenic stress can induce DNA damage and impact genomic stability. MYC-induced oxidative stress leads to oxidative DNA base damage [6]. In addition, MYC overexpression can also promote replication stress in cells [17]. Ataxia telangiectasia-mutated kinase (ATM) and ataxia telangiectasia and Rad3-related kinase (ATR) are critical for c-MYC-induced DNA damage response [18, 24, 32]. ATR is activated and recruited to sites of single-stranded (ss) double-stranded (ds) DNA damage, during nucleotide excision repair, at resected double-strand breaks and stalled replication forks. Activated ATR in turn phosphorylates Chk1 at Ser^345^ and Ser^317^, as well as several other target proteins involved in DNA repair and cell cycle progression [18, 24, 32]. ATM kinase is activated in response to DNA damage [34]. A key substrate of ATM is Chk2 whose phosphorylation at Thr68 results in activation and phosphorylation of a several proteins involved in DNA repair, recombination, cell cycle progression and apoptosis [34].

In the current study, we comprehensive investigated ATM, ATR and MYC expressions at the transcriptional levels (*n* = 1950) and at the protein level (*n* = 1650) breast tumours. We show that ATM and ATR levels have clinicopathological, predictive and prognostic significance in MYC overexpressed breast cancer.

## Methods

### Tissue culture and western blotting

Cell lines were purchased from American Type Culture Collection (ATCC, Manassas, USA). MDA-MB-231 and MDA-MB-468 cells were cultured in minimum essential amino acids medium supplemented with 1% L-glutamine and 1% non-essential amino acids. T47D cells were cultured in Dulbecco’s Modified Eagle’s medium. MCF-7 cells were grown in RPMI medium. All media were supplemented with 10% FBS and 1% penicillin streptomycin. Protein samples were prepared by lysing cells in RIPA buffer (Sigma–Aldrich) containing protease inhibitor (Sigma) and phosphatase inhibitor cocktail 1 and 2 (Sigma). Samples were run on SDS-PAGE gel (4–12%) bis-tris. Antibodies used were anti-MYC antibody (abcam, clone 9E10), ATM antibody (abcam clone Y170) and ATR antibody (cell signalling cat.no 2790S). Protein detection and quantification were determined by scanning the membranes on Licor-Odyssey’s Scanner (Licor, Biosciences) at the predefined intensity fluorescence.

### *MYC, ATM* and *ATR* mRNA expressions in breast cancer

*MYC, ATM* and *ATR* mRNA expressions were investigated in METABRIC (Molecular Taxonomy of Breast Cancer International Consortium) cohort. The METABRIC study protocol, detailing the molecular profiling methodology in a cohort of 1977 breast cancer samples is described by Curtis et al. [14]. Patient demographics are summarised in Supplementary Table S1 of supporting information. ER-positive and/or lymph node-negative patients did not receive adjuvant chemotherapy. ER-negative and/or lymph node-positive patients received adjuvant chemotherapy. For this cohort, the mRNA expression was hybridised to Illumina HT-12 v3 platform (Bead Arrays), and the data were pre-processed and normalised as described previously. Samples were classified into the intrinsic subtypes based on the PAM50 gene list. A description of the normalisation, segmentation and statistical analyses was previously described [14]. Real-time RT-qPCR was performed on the ABI Prism 7900HT sequence detection system (Applied Biosystems) using SYBR1 Green reporter. All the samples were analysed as triplicates. The Chi-square test was used for testing association between categorical variables, and a multivariate Cox model was fitted to the data using as endpoint breast cancer-specific death. X-tile (Version 3.6.1) was used to identify a cut-off in gene expression values such that the resulting subgroups had significantly different survival courses.

### MYC, ATM and ATR protein expressions in breast cancer

The study was performed in a consecutive series of 1650 patients with primary invasive breast carcinomas who were diagnosed between 1986 and 1999 and entered into the Nottingham Tenovus Primary Breast Carcinoma series. Patient demographics are summarised in Supplementary Table S2. This is a well-characterised series of patients with long-term follow-up that have been investigated in a wide range of biomarker studies [1, 2, 21]. All patients were treated in a uniform way in a single institution with standard surgery (mastectomy or wide local excision), followed by Radiotherapy. Prior to 1989, patients did not receive systemic adjuvant treatment (AT). After 1989, AT was scheduled based on prognostic and predictive factor status, including Nottingham Prognostic Index (NPI), oestrogen receptor-α (ER-α) status, and menopausal status. Patients with NPI scores of < 3.4 (low risk) did not receive AT. In pre-menopausal patients with NPI scores of ≥ 3.4 (high risk), classical Cyclophosphamide, Methotrexate, and 5-Fluorouracil (CMF), chemotherapy was given; patients with ER-α-positive tumours were also offered endocrine therapy. Postmenopausal patients with NPI scores of ≥ 3.4 and ER-α positivity were offered endocrine therapy, while ER-α-negative patients received classical CMF chemotherapy. Median follow-up was 111 months (range 1–233 months). Survival data, including breast cancer-specific survival (BCSS), disease-free survival (DFS), and development of loco-regional and distant metastases (DM), were maintained on a prospective basis. DFS was defined as the number of months from diagnosis to the occurrence of local recurrence, local lymph node (LN) relapse or DM relapse. Breast cancer-specific survival (BCSS) was defined as the number of months from diagnosis to the occurrence of BC-related death. Local recurrence-free survival (LRS) was defined as the number of months from diagnosis to the occurrence of local recurrence. DM-free survival was defined as the number of months from diagnosis to the occurrence of DM relapse. Survival was censored if the patient was still alive at the time of analysis, lost to follow-up, or died from other causes.

Tumour Marker Prognostic Studies (REMARK) criteria, recommended by McShane et al. [28], were followed throughout this study. Ethical approval was obtained from the Nottingham Research Ethics Committee (C202313).

### Tissue microarrays (TMAs) and immunohistochemistry (IHC)

Tumours were arrayed in tissue microarrays (TMAs) constructed with 0.6 mm cores sampled from the periphery of the tumours. The TMAs were immunohistochemically profiled for MYC, ATM and ATR and other biological antibodies as previously described [1, 2, 21]. Immunohistochemical staining was performed using the Thermo Scientific Shandon Sequenza chamber system (REF: 72110017), in combination with the Novolink Max Polymer Detection System (RE7280-K: 1250 tests), and the Leica Bond Primary Antibody Diluent (AR9352), each used according to the manufacturer’s instructions (Leica Microsystems). Leica Autostainer XL machine was used to dewax and rehydrate the slides. Pre-treatment antigen retrieval was performed on the TMA sections using sodium citrate buffer (pH 6.0) and heated for 20 min at 95 °C in a microwave (Whirpool JT359 Jet Chef 1000W). A set of slides were incubated for 18 h at 4 °C with the primary mouse monoclonal anti-ATM antibody, clone Y170 (Ab32420, Abcam, Cambridge, UK), at a dilution of 1:100. A set of slides were incubated for 18 h at 4 °C with the primary mouse monoclonal anti-ATR antibody, clone 1E9 (H00000545-M03, Novus Biologicals, Cambridge, UK), at a dilution of 1:20. A set of slides were incubated for 45 min at 4 °C with mouse monoclonal primary antibody for c-MYC (Clone 9E100; Abcam Ltd) at a dilution of 1: 100.

### Evaluation of immune staining

Whole field inspection of the core was scored and intensities of nuclear staining were grouped as follows: 0 = no staining, 1 = weak staining, 2 = moderate staining, 3 = strong staining. The percentage of each category was estimated (0–100%). H-score (range 0–300) was calculated by multiplying intensity of staining and percentage staining. X-tile (version 3.6.1, Yale University, USA) was used to identify a cut-off for ATM protein expression. The percentage of positive cells was used, with a cut-off of < 25% cells being classed as low, and ≥ 25% as high for ATM protein level. X-tile (version 3.6.1, Yale University, USA) was used to identify a cut-off for protein expression. H-score of ≥ 60 was taken as the cut-off for high ATR expression. Assessment of MYC staining was estimated subjectively on intensity corresponding to negative, weak, moderate and strong nuclear and/or cytoplasmic staining. Dichotomisation of c-MYC protein expression was based on the mean resulting in negative/weak (MYC negative) and moderate/strong (MYC positive) groups, which were selected prior to analysis.

### Statistical analysis

Data analysis was performed using SPSS (SPSS, version 17 Chicago, IL). Where appropriate, Pearson’s Chi-square, Fisher’s exact, Student’s t and ANOVA one-way tests were used. Cumulative survival probabilities were estimated using the Kaplan–Meier method, and differences between survival rates were tested for significance using the log-rank test. Multivariate analysis for survival was performed using the Cox proportional hazard model. The proportional hazards assumption was tested using standard log–log plots. Hazard ratios (HR) and 95% confidence intervals (95% CI) were estimated for each variable. All tests were two-sided with a 95% CI and a *p* value < 0.05 considered significant. For multiple comparisons, p values were adjusted according to Benjamini–Hochberg method [23].

## Results

**Fig. 1:**
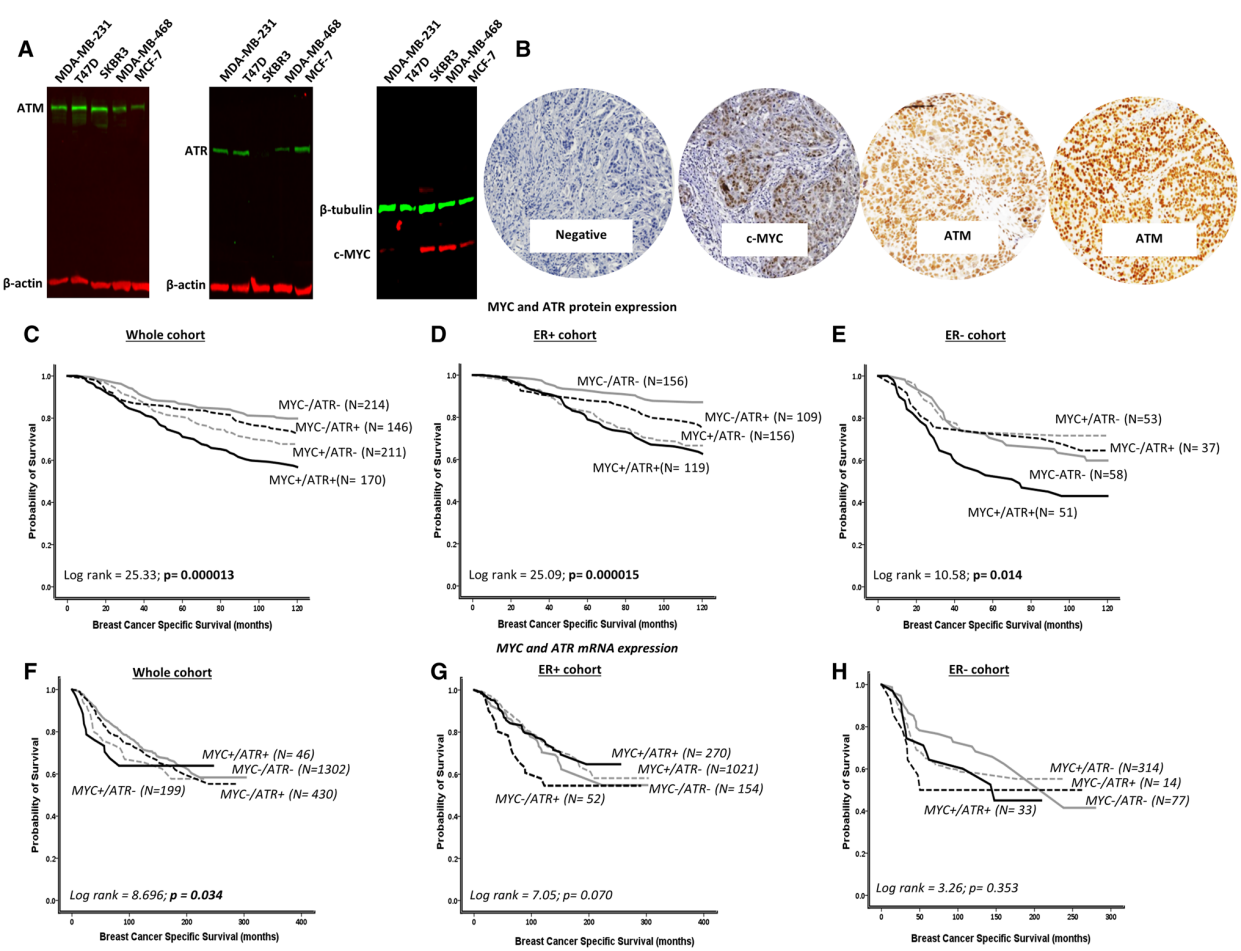
**a** Western blot of c-MYC, ATM and ATR expressions in breast cancer cell lines. **b** Microphotograph of MYC-negative and MYC-positive breast cancers. **c**–**h** Kaplan–Meier curves showing BCSS in MYC and ATR co-expression at protein and mRNA levels

We initially performed western blots in a panel of breast cancer cell lines to confirm the specificity of antibodies for IHC in the current study. As shown in Fig. 1a, the antibodies tested were not only specific but also demonstrated a spectrum of protein expression levels across various breast cancer cell lines (Supplementary Fig. S1). We then proceeded to investigate MYC, ATR and ATM protein levels in clinical breast carcinoma samples.

### High ATR promotes aggressive phenotypes in MYC overexpressed breast cancers

A total of 793 tumours were suitable for ATR and MYC protein co-expression analyses (Fig. 1b). Tumours with high MYC and high ATR expressions were significantly associated with vascular invasion, higher tumour grade, pleomorphism, high mitotic index and high-risk Nottingham Prognostic Index (NPI) (all adjusted *p* values ≤ 0.01) (Table 1).

**Table 1:** ATR and MYC protein co-expression in Sporadic Breast Cancer

Variable	ATR and MYC protein co-expression				*p* value	Adjusted *p* value*
	MYC−/ATR−	MYC−/ATR+	MYC+/ATR−	MYC+/ATR+		
	*N* (%)	*N* (%)	*N* (%)	*N* (%)		
Tumour size (cm)						
≤ 2.0	115 (50.0)	59 (38.1)	110 (49.1)	73 (39.7)	**0.03**	0.053
> 2.0	115 (50.0)	96 (61.9)	114 (50.9)	111 (60.3)		
Vascular invasion						
Negative	166 (72.5)	94 (60.6)	152 (68.8)	106 (57.6)	**0.005**	**0.013**
Positive	63 (27.5)	61 (39.4)	69 (31.2)	78 (42.4)		
Tumour grade^a^						
G1	48 (20.9)	18 (11.8)	40 (17.9)	15 (8.2)	**0.001**	**0.004**
G2	78 (33.9)	42 (27.4)	70 (31.4)	51 (27.9)		
G3	104 (45.2)	93 (60.8)	113 (50.7)	117 (63.9)		
Tumour type						
Ductal (incl mixed)	192 (83.6)	134 (86.4)	195 (87.1)	169 (91.8)	0.076	0.121
Lobular	20 (8.7)	15 (9.7)	13 (5.8)	7 (3.8)		
Medullary-like	7 (3.0)	0 (0.0)	10 (4.5)	6 (3.3)		
Miscellaneous	1 (0.4)	0 (0.0)	0 (0.0)	0 (0.0)		
Special type	10 (4.3)	6 (3.9)	6 (2.6)	2 (1.1)		
Tubules						
1	14 (6.4)	5 (3.2)	9 (4.3)	5 (2.7)	0.583	0.717
2	75 (34.4)	48 (31.4)	72 (34.1)	65 (35.5)		
3	129 (59.2)	100 (65.4)	130 (61.6)	113 (61.8)		
Pleomorphism						
1	2 (0.9)	1 (0.6)	6 (2.9)	1 (0.5)	**0.003**	**0.0096**
2	93 (42.7)	61 (39.9)	78 (37.3)	47 (25.7)		
3	123 (56.4)	91 (59.5)	125 (59.8)	135 (73.8)		
Mitosis						
1	92 (42.2)	40 (26.2)	66 (31.3)	38 (20.8)	**0.00012**	**0.00064**
2	36 (16.5)	21 (13.7)	40 (18.9)	35 (19.1)		
3	90 (41.3)	92 (60.1)	105 (49.8)	110 (60.1)		
NPI group						
GPG	79 (34.3)	35 (22.6)	71 (31.7)	33 (17.9)	< **0.00001**	< **0.00001**
MPG	128 (55.7)	83 (53.5)	125 (55.8)	96 (52.2)		
PPG	23 (10.0)	37 (23.9)	28 (12.5)	55 (29.9)		
ER status						
Negative	63 (27.4)	38 (24.5)	58 (26.2)	51 (27.7)	0.908	0.968
Positive	167 (72.6)	117 (75.5)	163 (73.8)	133 (72.3)		
PR status						
Negative	105 (45.9)	64 (42.4)	98 (44.7)	77 (42.3)	0.864	0.987
Positive	124 (54.1)	87 (57.6)	121 (55.3)	105 (57.7)		
HER2 status						
Negative	187 (82.7)	122 (81.3)	194 (87.0)	151 (82.5)	0.437	0.582
Positive	39 (17.3)	28 (18.7)	29 (13.0)	32 (17.5)		
Triple negative						
Non-triple negative	188 (82.5)	132 (86.8)	175 (79.5)	144 (78.3)	0.183	0.266
Triple negative	40 (17.5)	20 (13.2)	45 (20.5)	40 (21.7)		
Basal phenotype						
Negative	175 (76.8)	121 (78.1)	149 (66.8)	127 (69.0)	**0.027**	0.054
Positive	53 (23.2)	34 (21.9)	74 (33.2)	57 (31.0)		

Bold statistically significant; *NPI* Nottingham Prognostic Index, *GPG* Good Prognosis Group, *MPG* Moderate Prognosis Group, *PPG* Poor Prognosis Group, *HER2* human epidermal growth factor 2, *ER* oestrogen receptor, *PR* progesterone receptor

*Adjusted *P* value—Benjamini and Hochberg false discovery rate

^a^Grade as defined by Nottingham Grading System

At the transcriptomic level (Table 2), tumours with high *MYC* mRNA and high *ATR* mRNA expression were also associated with higher tumour grade, high-risk Nottingham Prognostic Index (NPI), ER−, PR−, Genefu subtype (ER−/Her-2−), triple negative and PAM50.Basal phenotypes (all adjusted *p* values ≤ 0.01). Interestingly, Genufu subtype (ER+/HER-2−/low proliferation), Her-2+, PAM50.Her-2 subtype and PAM50.Luminal A subtype were more common in tumours with low *MYC* mRNA and low *ATR* mRNA expressions (all adjusted *p* values ≤ 0.01).

**Table 2:** ATR and MYC mRNA co-expression in sporadic breast cancer

Variable	*ATR* and *MYC* mRNA co-expression				*p* value	Adjusted *p* value*
	MYC−/ATR−	MYC−/ATR+	MYC+/ATR−	MYC+/ATR+		
	*N* (%)	*N* (%)	*N* (%)	*N* (%)		
Tumour size (cm)						
T 1a+b(1.0)	58 (4.5)	24 (5.6)	9 (4.6)	1 (2.2)	0.333	0.428
T 1c(> 1.0–2.0)	527 (40.9)	156 (36.6)	64 (32.5)	19 (42.2)		
T2 (> 2.0–5)	648 (50.3)	220 (51.6)	111 (56.3)	22 (48.9)		
T3 (> 5)	56 (4.3)	26 (6.1)	13 (6.6)	3 (6.7)		
Lymph node stage						
Negative	686 (52.8)	232 (54.1)	91 (46.4)	26 (56.5)	0.612	0.718
Positive (1–3)	210 (16.2)	65 (15.2)	34 (17.3)	5 (10.9)		
Positive (> 3)	404 (31.1)	132 (30.8)	71 (36.2)	15 (32.6)		
Grade^a^						
G1	120 (9.7)	37 (9.0)	11 (5.6)	1 (2.3)	**0.00018**	**0.00048**
G2	524 (42.2)	176 (42.9)	57 (29.2)	13 (30.2)		
G3	597 (48.1)	197 (48.0)	127 (65.1)	29 (67.4)		
NPI						
≤ 3.4	274 (22.2)	84 (20.7)	25 (13.2)	5 (11.9)	**0.016**	**0.0288**
> 3.4	958 (77.8)	322 (79.3)	165 (86.8)	37 (88.1)		
HER 2 overexpression						
No	1119(85.9)	382 (88.8)	187 (94.0)	44 (95.7)	**0.003**	**0.00675**
Yes	183 (14.1)	48 (11.2)	12 (6.0)	2 (4.3)		
ER						
Negative	270 (20.7)	92 (21.4)	84 (42.2)	24 (52.2)	< **0.00001**	< **0.00001**
Positive	1032(79.3)	338 (78.6)	115 (57.8)	22 (47.8)		
PR						
Negative	597 (45.9)	194 (45.1)	116 (58.3)	29 (63.0)	**0.001**	**0.00245**
Positive	705 (54.1)	236 (54.9)	83 (41.7)	17 (37.0)		
Triple negative						
No	1137(87.3)	371 (86.3)	126 (63.3)	26 (56.5)	< **0.00001**	< **0.00001**
Yes	165 (12.7)	59 (13.7)	73 (36.7)	20 (43.5)		
Genefu subtype						
ER−/Her-2 negative	77 (11.5)	30 (14.6)	35 (32.4)	8 (57.1)	< **0.00001**	< **0.00001**
ER+/Her-2 negative/high proliferation	231 (34.6)	87 (42.4)	45 (41.7)	3 (21.4)	0.083	0.124
ER+/Her-2 negative/low proliferation	280 (42.0)	65 (31.7)	21 (19.4)	2 (14.3)	**0.00001**	**0.00002**
Her-2 positive	79 (11.8)	23 (11.2)	7 (6.5)	1 (7.1)	0.401	0.492
PAM50 subtype						
PAM50.Her2	166 (14.2)	66 (16.4)	6 (3.7)	0 (0.0)	**0.00005**	**0.000156**
PAM50.Basal	173 (14.8)	54 (13.4)	77 (47.0)	26 (74.3)	< **0.00001**	< **0.00001**
PAM50.LumA	509 (43.5)	166 (41.2)	38 (23.2)	2 (5.7)	< **0.00001**	< **0.00001**
PAM50.LumB	322 (27.5)	117 (29.0)	43 (26.2)	7 (20.0)	0.663	0.745

Bold statistically significant; *NPI* Nottingham Prognostic Index, *HER2* human epidermal growth factor 2, *ER* oestrogen receptor, *PR* progesterone receptor

*Adjusted *P* value—Benjamini and Hochberg false discovery rate

^a^Grade as defined by Nottingham Grading System

**Fig. 2:**
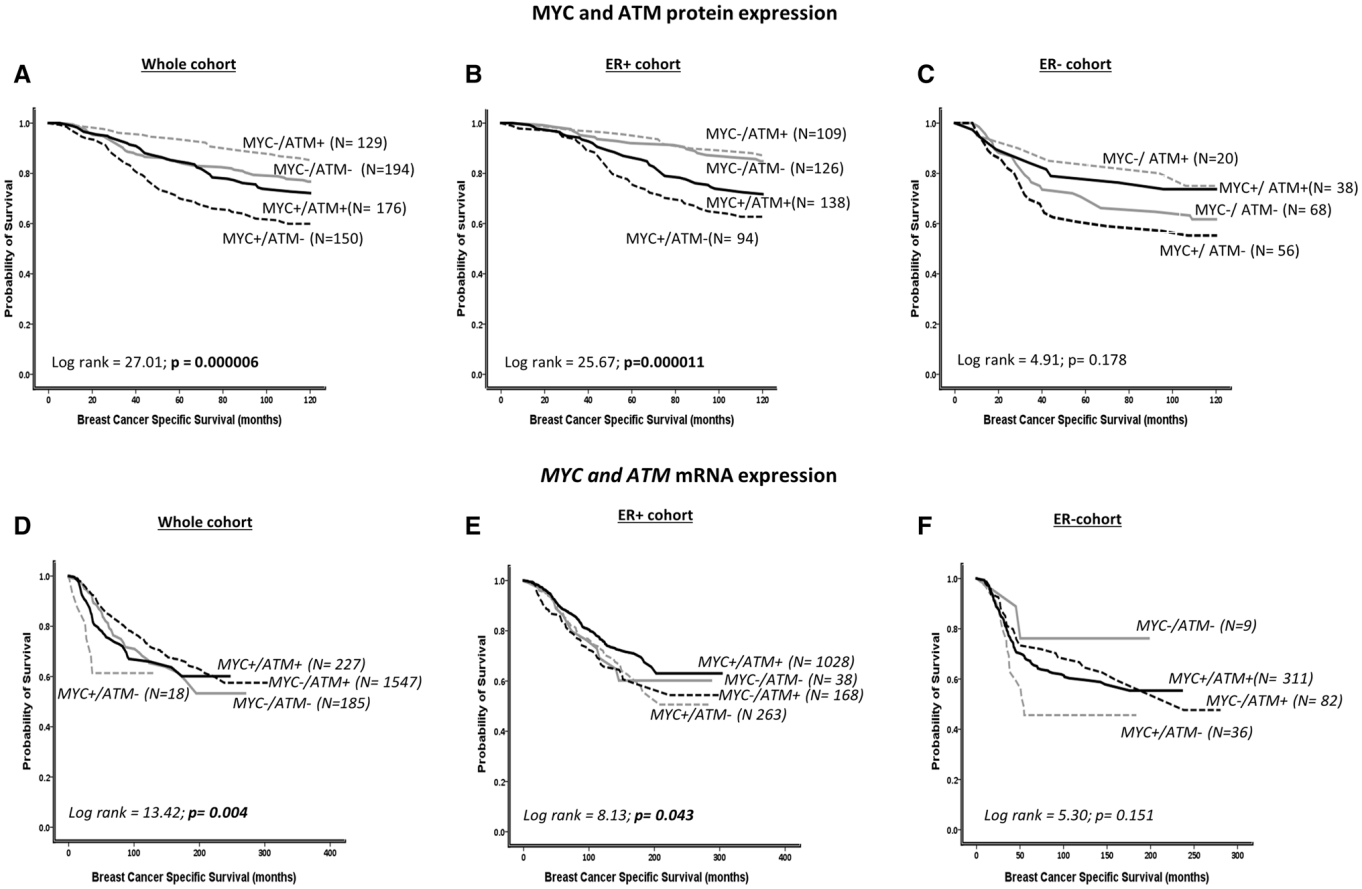
Kaplan–Meier curves showing BCSS in MYC and ATM co-expression at protein and mRNA levels

We then investigated the prognostic significance of MYC-ATR co-expression in breast cancers. In the whole cohort, as shown in Fig. 1c, f, patients with MYC overexpressed tumours and high ATR protein or mRNA expression had a worse overall breast cancer-specific survival (BCSS) (*p* < 0.001). In ER + breast cancer, similarly, MYC overexpressed tumours with high ATR levels are associated with worse survival (*p* < 0.001) (Fig. 1d) including in patients who received endocrine therapy (Supplementary Figs. S2B and S2F). In ER- tumours, MYC overexpressed tumours with high ATR protein levels had the worst survival (Fig. 2e). Together the data show that MYC-ATR co-expression has prognostic significance in breast cancers.

### Low ATM promotes aggressive phenotypes in MYC overexpressed breast cancers

A total of 696 tumours were suitable for ATM and MYC protein expression analyses (Fig. 1b) (Table 3). Tumours with high MYC and low ATM expressions were significantly associated with higher tumour grade, tumour type, pleomorphism, high mitotic index, ER−, PR−, triple negative, basal phenotypes and high-risk Nottingham Prognostic Index (NPI) (all adjusted *p* values ≤ 0.001).

**Table 3:** ATM and MYC protein co-expression in sporadic breast cancer

Variable	ATM and MYC protein co-expression				*p* value	Adjusted *p* value*
	MYC−/ATM−	MYC−/ATM+	MYC+/ATM−	MYC+/ATM+		
	*N* (%)	*N* (%)	*N* (%)	*N* (%)		
Tumour size (cm)						
≤ 2.0	93 (45.8)	77 (54.2)	66 (41.0)	96 (50.5)	0.101	1.616
> 2.0	110 (54.2)	65 (45.8)	95 (59.0)	94 (49.5)		
Vascular invasion						
Negative	127 (62.9)	112 (78.9)	103 (64.0)	129 (68.3)	**0.01**	**0.013**
Positive	75 (37.1)	30 (21.1)	58 (36.0)	60 (31.7)		
Tumour grade^a^						
G1	23 (11.3)	39 (27.2)	9 (5.6)	41 (21.7)	< **0.00001**	< **0.00001**
G2	61 (30.0)	54 (37.8)	48 (29.8)	60 (31.7)		
G3	119 (58.6)	50 (35.0)	104 (64.6)	88 (46.6)		
Tumour type						
Ductal (incl mixed)	172 (84.7)	117 (82.4)	140 (87.0)	169 (89.0)	**0.023**	**0.028**
Lobular	17 (8.5)	18 (12.7)	10 (6.2)	9 (4.7)		
Medullary-like	7 (3.4)	0 (0.0)	9 (5.6)	8 (4.2)		
Special type	7 (3.4)	7 (4.9)	2 (1.2)	4 (2.1)		
Tubules						
1	9 (4.5)	10 (7.1)	5 (3.1)	6 (3.2)	**0.00016**	**0.00028**
2	49 (24.6)	57 (40.7)	45 (28.7)	83 (44.9)		
3	141 (70.9)	73 (52.2)	107 (68.2)	96 (51.9)		
Pleomorphism						
1	4 (2.0)	4 (2.8)	1 (0.7)	6 (3.3)	**0.00007**	**0.00015**
2	69 (34.7)	74 (52.9)	41 (26.1)	73 (39.9)		
3	126 (66.3)	62 (44.3)	115 (73.2)	104 (56.8)		
Mitosis						
1	58 (29.1)	75 (53.6)	29 (18.5)	62 (33.5)	< **0.00001**	< **0.00001**
2	35 (17.6)	17 (12.1)	28 (17.8)	42 (22.7)		
3	106 (53.3)	48 (34.3)	100 (63.7)	81 (43.8)		
NPI group						
GPG	48 (23.7)	59 (41.6)	28 (17.4)	65 (34.2)	< **0.00001**	**0.00001**
MPG	118 (58.1)	76 (53.5)	97 (60.2)	100 (52.6)		
PPG	37 (18.2)	7 (4.9)	36 (22.4)	25 (13.2)		
ER status						
Negative	69 (33.8)	23 (16.1)	59 (36.6)	40 (21.1)	**0.00002**	**0.00007**
Positive	135 (66.2)	120 (83.9)	102 (63.4)	150 (78.9)		
PR status						
Negative	100 (49.8)	41 (29.1)	83 (51.6)	65 (34.4)	**0.00001**	**0.00006**
Positive	101 (50.2)	100 (70.9)	78 (48.4)	124 (65.6)		
HER2 status						
Negative	163 (81.5)	127 (91.4)	136 (85.0)	158 (83.2)	0.081	0.086
Positive	37 (18.5)	12 (8.6)	24 (15.0)	32 (16.8)		
Triple negative						
Non-triple negative	159 (79.1)	124 (87.3)	112 (69.6)	165 (86.8)	**0.00007**	**0.00015**
Triple negative	42 (20.9)	18 (12.7)	49 (30.4)	25(13.2)		
Basal phenotype						
Negative	160 (79.6)	110 (78.6)	107 (66.5)	131 (68.9)	**0.008**	**0.0116**
Positive	41 (20.3)	30 (21.4)	54 (33.5)	59 (30.1)		

Bold statistically significant; *NPI* Nottingham Prognostic Index, *GPG* Good Prognosis Group, *MPG* Moderate Prognosis Group, *PPG* Poor Prognosis Group, *HER2* Human epidermal growth factor 2, *ER* oestrogen receptor, *PR* progesterone receptor; Basal-like: ER−, HER2− and positive expression of either CK5/6, CK14, or EGFR; Triple negative: ER−/PR−/HER2−

*Adjusted *p* value—Benjamini and Hochberg false discovery rate

^a^Grade as defined by Nottingham Grading System

At the transcriptomic level (Table 4), tumours with high *MYC* mRNA and low *ATM* mRNA expressions had increased tumour size, ER− and PR− tumours (all adjusted *p* values ≤ 0.01). Interestingly, Genufu subtype (ER+/HER-2−/low proliferation) and PAM50.Luminal A subtype were common in tumours with low *MYC* mRNA and high ATM mRNA expressions all adjusted *p* values ≤ 0.01). Her-2+ and PAM50.Her-2 subtypes were more common in tumours with low *MYC* mRNA and low *ATM* mRNA expressions (all adjusted *p* values ≤ 0.01). Whereas triple negative, Genefu subtype (ER−/Her-2−), PAM50.Basal were frequently expressing in tumours with high *MYC* mRNA and high *ATM* mRNA expressions (all adjusted *p* values ≤ 0.01).

**Table 4:** *ATM* and *MYC* mRNA co-expression in sporadic breast cancer

Variable	*ATM* and *MYC* mRNA co-expression				*p* value	Adjusted *p* value*
	MYC−/ATM−	MYC−/ATM+	MYC+/ATM−	MYC+/ATM+		
	*N* (%)	*N* (%)	*N* (%)	*N* (%)		
Tumour size (cm)						
T 1a+b(1.0)	6 (3.3)	76 (5.0)	3 (16.7)	7 (3.1)	**0.00038**	**0.0009**
T 1c(> 1.0–2.0)	72 (39.3)	611 (39.9)	2 (11.1)	81 (36.2)		
T2 (> 2.0–5)	89 (48.6)	779 (50.8)	9 (50.0)	124 (55.4)		
T3 (> 5)	16 (8.7)	66 (4.3)	4 (22.2)	12 (5.4)		
Lymph node stage						
Negative	98 (53.0)	820 (53.1)	9 (50.0)	108 (48.2)	0.803	
Positive (1–3)	32 (17.3)	243 (15.7)	2 (11.1)	37 (16.5)		
Positive (> 3)	55 (29.7)	481 (31.2)	7 (38.9)	79 (35.3)		
Grade^a^						
G1	20 (11.2)	137 (9.3)	0 (0.0)	12 (5.4)	**0.00006**	**0.0002**
G2	73 (40.8)	627 (4.6)	8 (47.1)	62 (28.1)		
G3	86 (48.0)	708 (48.1)	9 (52.9)	147 (66.5)		
NPI						
≤ 3.4	36 (20.3)	322 (22.0)	3 (17.6)	27 (12.6)	**0.016**	**0.0288**
> 3.4	141 (79.7)	1139 (78.0)	14 (82.4)	188 (87.4)		
HER 2 overexpression						
No	157 (84.9)	1344 (86.9)	17 (94.4)	214 (94.3)	**0.007**	**0.01**
Yes	28 (15.1)	203 (13.1)	1 (5.6)	13 (5.7)		
ER						
Negative	39 (21.1)	323 (20.9)	6 (33.3)	102 (44.9)	< **0.00001**	< **0.00001**
Positive	146 (78.9)	1224 (79.1)	12 (66.7)	125 (55.1)		
PR						
Negative	76 (41.1)	715 (46.2)	14 (77.8)	131 (57.7)	**0.00016**	**0.0004**
Positive	109 (58.9)	832 (53.8)	4 (22.2)	96 (42.3)		
Triple negative						
No	162 (87.6)	1346 (87.0)	13 (72.2)	139 (61.2)	< **0.00001**	< **0.00001**
Yes	23 (12.4)	201 (13.0)	5 (27.8)	88 (38.8)		
Genefu subtype						
ER−/Her-2 negative	12 (12.9)	95 (12.2)	2 (25.0)	41 (36.0)	< **0.00001**	< **0.00001**
ER+/Her-2 negative/high proliferation	33 (35.5)	285 (36.6)	3 (37.5)	45 (39.5)	0.933	25.191
ER+/Her-2 negative/low proliferation	34 (36.6)	311 (39.9)	2 (25.0)	21 (18.4)	**0.00015**	**0.0004**
Her-2 positive	14 (15.1)	88 (11.3)	1 (12.5)	7 (6.1)	0.224	0.288
PAM50 subtype						
PAM50.Her2	31 (18.3)	201 (14.3)	1 (7.1)	5 (2.7)	**0.00004**	**0.0001**
PAM50.Basal	23 (13.6)	204 (14.5)	6 (42.9)	97 (52.4)	< **0.00001**	< **0.00001**
PAM50.LumA	67 (39.6)	608 (43.3)	2 (14.3)	38 (20.5)	< **0.00001**	< **0.00001**
PAM50.LumB	48 (28.4)	391 (27.8)	5 (35.7)	45 (24.3)	0.67	0.7865

Bold statistically significant; *NPI* Nottingham Prognostic Index, *HER2* Human epidermal growth factor 2, *ER* oestrogen receptor, *PR* progesterone receptor

*Adjusted *p* value—Benjamini and Hochberg false discovery rate

^a^Grade as defined by Nottingham Grading System

We then investigated the prognostic significance of MYC-ATM co-expression in breast cancers. In the whole cohort, as shown in Fig. 2a, d, patients with MYC overexpressed tumours with low ATM protein or mRNA expression had worse overall breast cancer-specific survival (BCSS) (*p* < 0.001). In ER+ breast cancer, similarly, MYC overexpressed tumours with low ATM levels were associated with worse survival (*p* < 0.001) (Fig. 2b, e) including in patients who received endocrine therapy (Supplementary Figs. S3B and S3F). In ER− tumours that received no chemotherapy, MYC overexpressed tumours with low ATM protein levels had the worst survival (Supplementary Fig. S3C). Whereas at the mRNA level, ER− tumours that received chemotherapy have poor survival (*p* = 0.028) (Supplementary Fig. S3H). Together, our data show that MYC-ATM co-expression has prognostic significance in breast cancers.

## Discussion

Oxidative and oncogenic stresses in MYC overexpressed tumours will induce DNA damage. The DNA damage signalling proteins, ATR and ATM, are critical for the maintenance of genomic instability [6, 15]. Although MYC amplification promotes aggressive breast cancer phenotype [7, 16, 21, 38], whether ATR and ATM expressions influence pathology and clinical outcomes in MYC overexpressed breast cancers is unknown.

MYC promotes cellular proliferation by several mechanisms, including by promoting replication and transcriptional response [5, 15, 17]. However, MYC overexpression also induces replication stress [17]. Activation of ATR-mediated signalling is a key compensatory response to mitigate replication stress in MYC overexpressed tumours [6]. Therefore, ATR overexpression in MYC overexpressed tumours will be expected to promote proliferation and aggressive phenotypes. In the current study, we provide the first clinical evidence that high ATR in MYC overexpressed tumours is associated with aggressive cancer and poor survival. Although direct targeting MYC for cancer therapy has been challenging [9, 26], the clinical data shown here would suggest that ATR-Chk1 pathway targeting could be an alternative anti-cancer approach in MYC-amplified breast cancers. A previous preclinical study investigating the role of Chk1 expression in MYC amplified tumours has in fact shown that its blockade resulted in caspase-depended apoptosis of the MYC-overexpressing tumours cells both in vitro and in murine models of B-cell lymphoma [22]. MYC is a well-known ER-regulated gene and its overexpression is linked to resistance to endocrine therapy [7, 8, 21, 33, 36, 39]. In addition, MYC is frequently overexpressed during progression and distant relapse of ER+ breast cancers and predicts poor outcome following adjuvant endocrine treatment [21, 29]. In another study, MYC expression was up-regulated in aromatase inhibitor-resistant breast cancer cells and reduction of MYC expression significantly decreased cell proliferation in breast cancer cell lines [8]. In the current study, high ATR in MYC overexpressed tumours was linked to poor survival particularly in patients who received endocrine therapy providing further evidence for ATR as a predictive factor in MYC overexpressed ER+ breast cancers. As ATR inhibition is a promising anti-cancer approach [18, 24], whether combining ATR inhibitor with endocrine therapy in MYC amplified tumours will be clinically relevant will be an interesting area for future investigation.

Proficient ATM-mediated pathways act as robust anti-cancer barriers [3, 4, 10, 11, 13]. In contrast, ATM deficiency either in the germ-line or due to epigenetic mechanisms is well known to increase cancer risk and promote breast cancers [3, 4, 10, 11, 13]. For example, ATM has been shown to promote apoptosis and suppress tumorigenesis in response to MYC [31]. Therefore, ATM deficiency in MYC overexpressed breast cancer will be expected to promote aggressive breast cancers. As expected, in contrast to ATR, we observed that low ATM was linked to aggressive phenotypes in MYC overexpressed breast cancers, including in ER+ tumours. PARP [20] or ATR inhibition [25, 30] can induce synthetic lethality in ATM-deficient haematological malignancies. Therefore, it will be important to evaluate if a similar approach could be employed to personalise therapy in ATM-deficient MYC overexpressed breast cancers.

In conclusion, we provide strong clinical evidence that ATM signalling and ATR signalling can influence clinicopathological features and survival outcomes in patients with MYC overexpressed breast cancer.

## Electronic supplementary material

Below is the link to the electronic supplementary material.

Supplementary material 1 (DOCX 20 KB)

Supplementary material 2 (DOCX 13 KB)

Supplementary material 3 (TIF 157 KB)

Supplementary material 4 (TIF 741 KB)

Supplementary material 5 (TIF 744 KB)
