# Supplementary material for: Clinicopathological significance of ataxia telangiectasia-mutated (ATM) kinase and ataxia telangiectasia-mutated and Rad3-related (ATR) kinase in MYC overexpressed breast cancers
Source: Breast Cancer Res Treat. 2019 Feb 12;175(1):105–15. doi: 10.1007/s10549-018-05113-8 (PMC6491658; doi:10.1007/s10549-018-05113-8)
Supplement: Supplementary file 1 — Supplementary material 1 (DOCX 20 KB) [file 10549_2018_5113_MOESM1_ESM.docx]

**Supplementary Table S1:** Clinicopathological characteristics in the METABRIC cohort

| **Variables** | **N (%)** |
| --- | --- |
| **Age at diagnosis** [Median (range)] | 61.8 (21.93-96.29) |
| **Tumour size** [Median (range)] | 23 (1, 182) |
| **NPI** [Median (95% CI)] | 4.46 (4.41-4.51) |
| **Survival** [Median (Months, 95% Cl)] | 149 (141-159) |
| **Lymph nodes status**  0  1  2  3  >3 | 1035  337  171  114  314 |
| **ER status**  Positive  Negative  Null | 1497  438  42 |
| **PAM50 subtype**  Basal  HER2  Luminal A  Luminal B  Normal  Not classified | 330  238  715  489  199  6 |
| **Adjuvant systemic therapy (AT**) |  |
| No AT | 305 |
| Hormone therapy (HT) | 1216 |
| Chemotherapy | 416 |
| Hormone + chemotherapy | 192 |

**Supplementary Table S2:** Clinicopathological characteristics of Nottingham series

| **Variable** | **n*** | **Cases (%)** |
| --- | --- | --- |
| **Menopausal status** | 1650 |  |
| Pre-menopausal |  | 612 (37.0) |
| postmenopausal |  | 1038 (63.0) |
| **Tumour Grade (NGS)** | 1650 |  |
| G1 |  | 306 (18.5) |
| G2 |  | 531 (32.2) |
| G3 |  | 813 (49.3) |
| **Lymph node stage** | 1650 |  |
| Negative |  | 1056 (64.0) |
| Positive (1-3 nodes) |  | 486 (29.5) |
| Positive (>3 nodes) |  | 108 (6.5) |
| **Tumour size (cm)** | 1650 |  |
| T1 a + b (≤1.0) |  | 187 (11.0) |
| T1 c (>1.0 -2.0) |  | 868 (53.0) |
| T2 (>2.0-5) |  | 579 (35.0) |
| T3 (>5) |  | 16 (1.0) |
| **Tumour type** | 1650 |  |
| IDC-NST |  | 941 (57) |
| Tubular |  | 349 (21) |
| ILC |  | 160 (10) |
| Medullary (typical/atypical) |  | 41 (2.5) |
| Others |  | 159 (9.5) |
| **NPI subgroups** | 1650 |  |
| Excellent PG(2.08-2.40) | Low risk | 207 (12.5) |
| Good PG(2.42-3.40) |  | 331 (20.1) |
| Moderate I PG(3.42 to 4.4) | High risk | 488 (29.6) |
| Moderate II PG(4.42 to 5.4) |  | 395 (23.9) |
| Poor PG(5.42 to 6.4) |  | 170 (10.3) |
| Very poor PG(6.5–6.8) |  | 59 (3.6) |
| **Survival at 20 years** | 1650 |  |
| Alive and well |  | 1055 (64.0) |
| Dead from disease |  | 468 (28.4) |
| Dead from other causes |  | 127 (7.6) |
| **Adjuvant systemic therapy (AT**) |  |  |
| No AT |  | 665 (42.0) |
| Hormone therapy (HT) |  | 642 (41.0) |
| Chemotherapy |  | 307 (20.0) |
| Hormone + chemotherapy |  | 46 (3.0) |

* Number of cases for which data were available.

NPI; Nottingham prognostic index, PG; prognostic group
