# Supplementary material for: Clinicopathological significance of ataxia telangiectasia-mutated (ATM) kinase and ataxia telangiectasia-mutated and Rad3-related (ATR) kinase in MYC overexpressed breast cancers
Source: Breast Cancer Res Treat. 2019 Feb 12;175(1):105–15. doi: 10.1007/s10549-018-05113-8 (PMC6491658; doi:10.1007/s10549-018-05113-8)
Supplement: Supplementary file 2 — Supplementary material 2 (DOCX 13 KB) [file 10549_2018_5113_MOESM2_ESM.docx]

**Supplementary Figure legends**

**Supplementary Figure 1:** Protein quantification data.

**Supplementary Figure 2**. Kaplan-Meier curves showing BCSS in MYC and ATR co-expression with or without adjuvant treatment.

**Supplementary Figure 3**. Kaplan-Meier curves showing BCSS in MYC and ATM co-expression with or without adjuvant treatment.
