# Supplementary figures and images for: Clinicopathological significance of ataxia telangiectasia-mutated (ATM) kinase and ataxia telangiectasia-mutated and Rad3-related (ATR) kinase in MYC overexpressed breast cancers
Source: Breast Cancer Res Treat. 2019 Feb 12;175(1):105–15. doi: 10.1007/s10549-018-05113-8 (PMC6491658; doi:10.1007/s10549-018-05113-8)

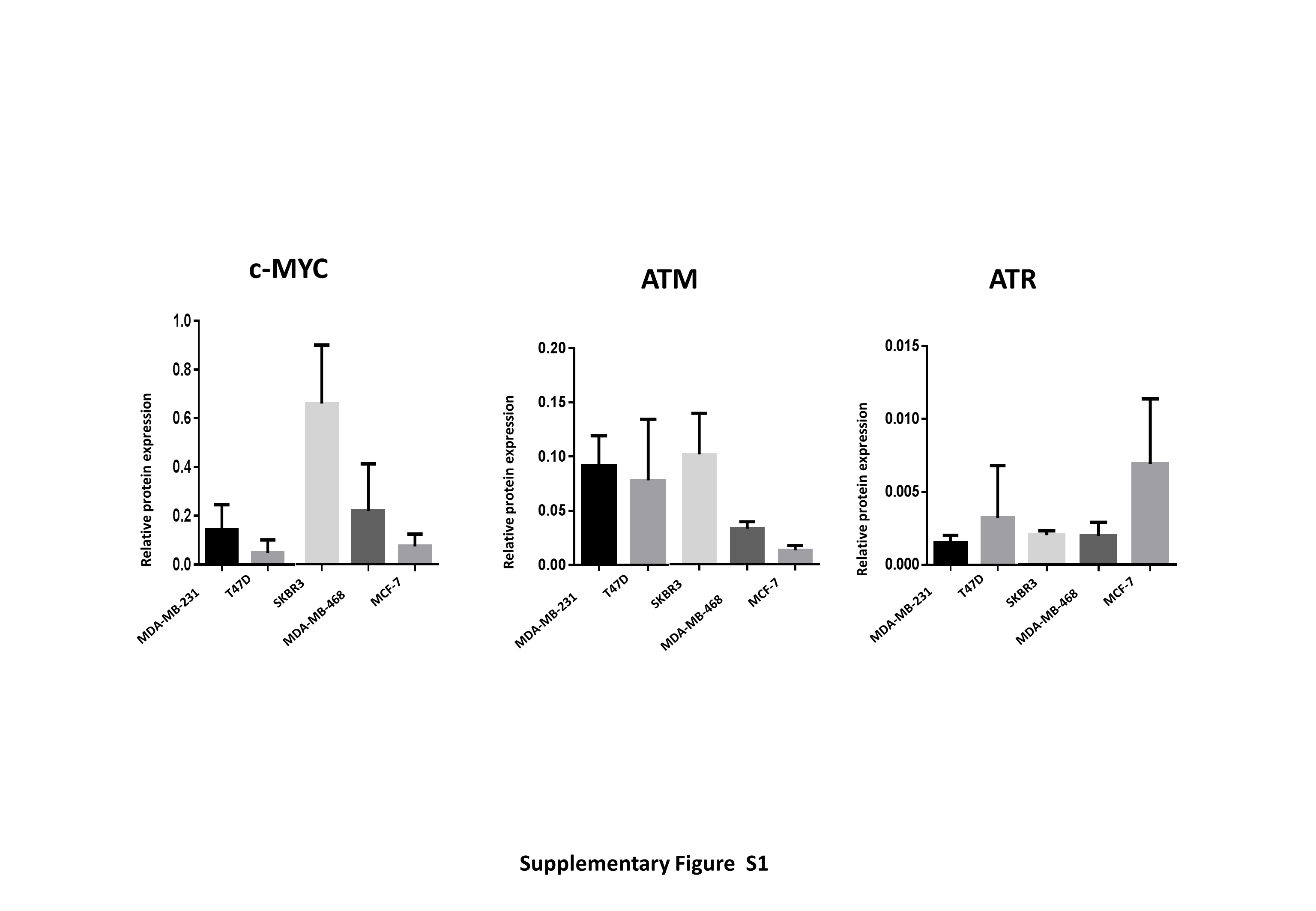

Supplement: Supplementary file 3 — Supplementary material 3 (TIF 157 KB) [file 10549_2018_5113_MOESM3_ESM.tif]

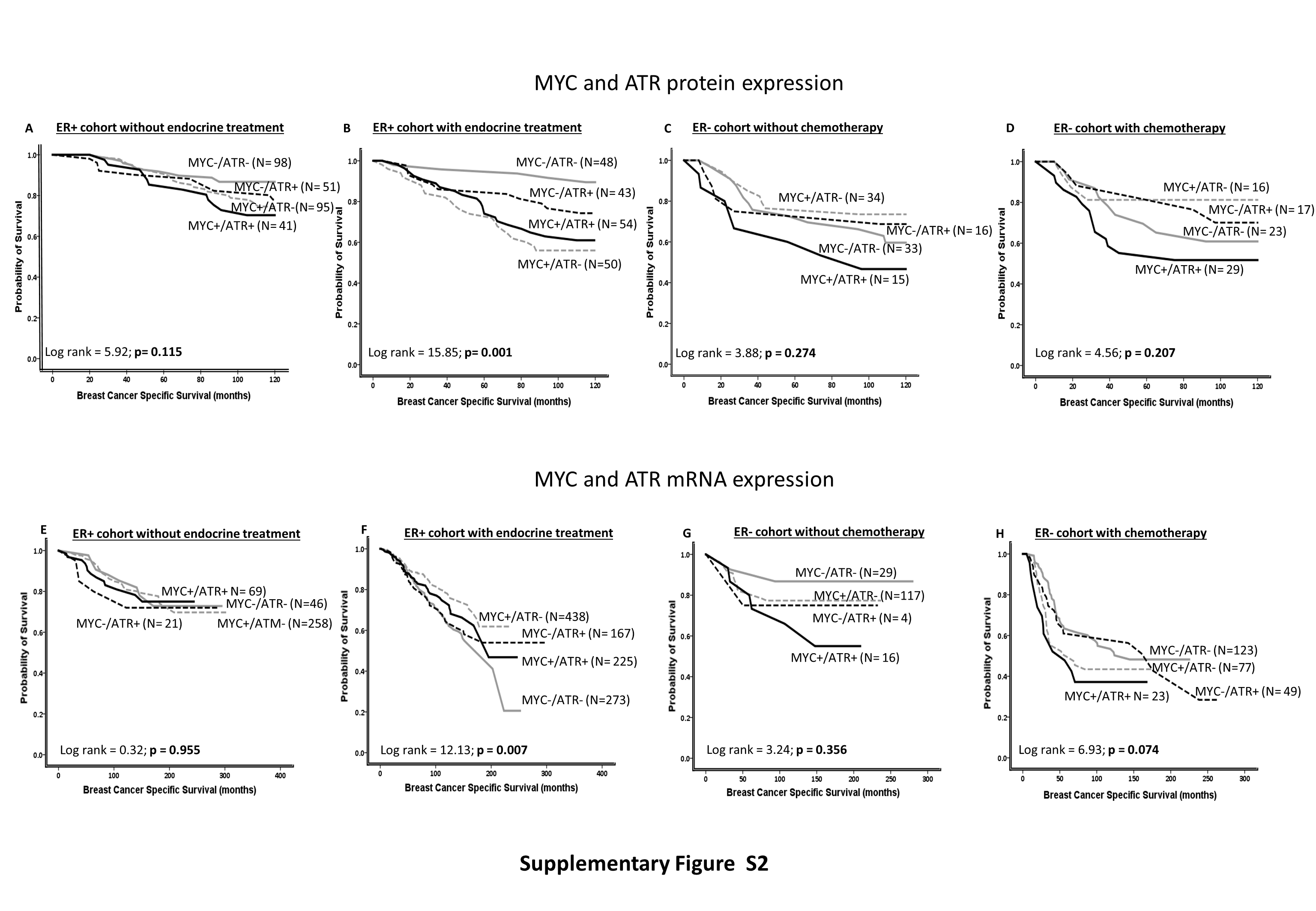

Supplement: Supplementary file 4 — Supplementary material 4 (TIF 741 KB) [file 10549_2018_5113_MOESM4_ESM.tif]

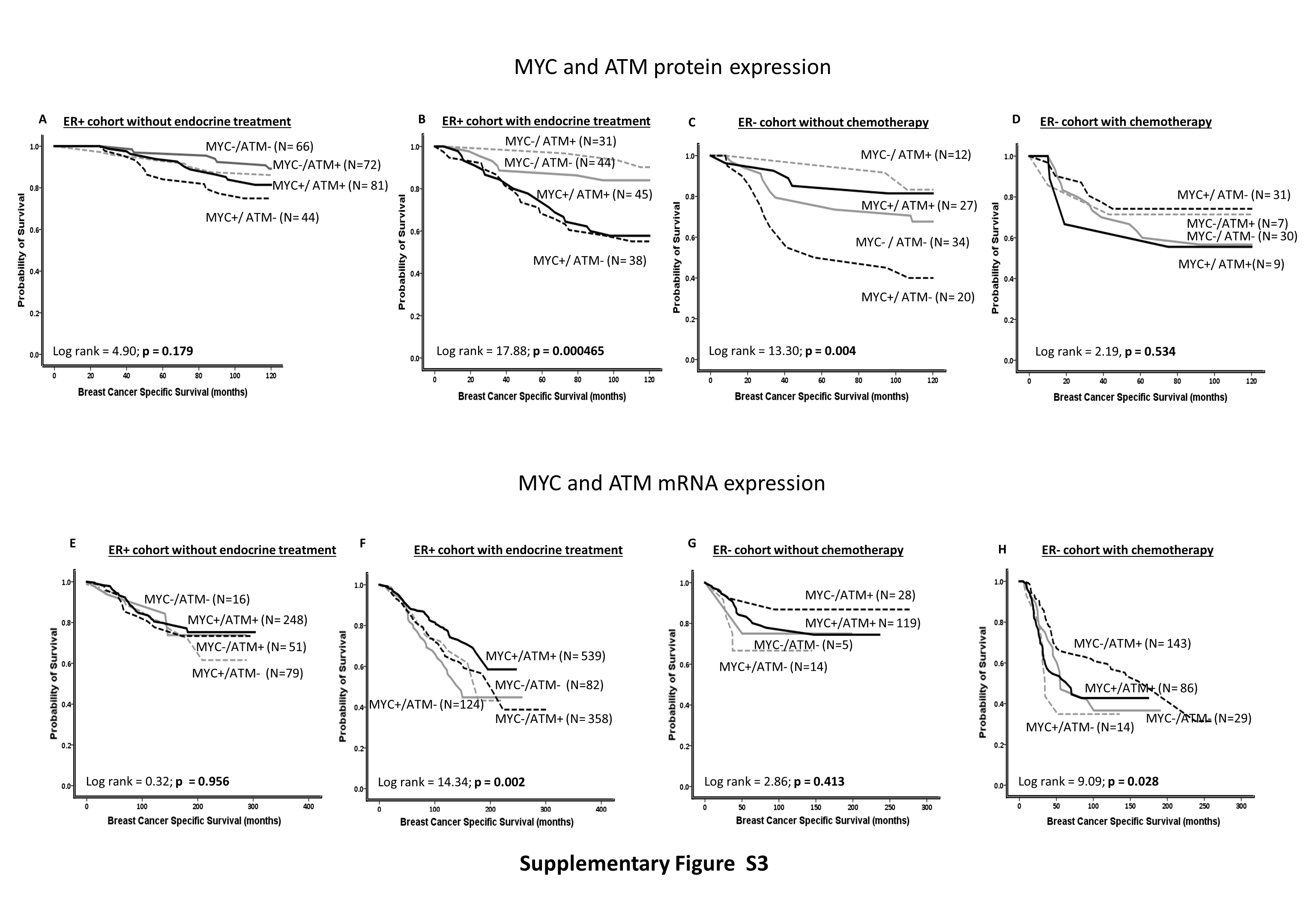

Supplement: Supplementary file 5 — Supplementary material 5 (TIF 744 KB) [file 10549_2018_5113_MOESM5_ESM.tif]
